# Recurrent Intestinal Angioedema with Normal C1-Inhibitor: A Case Report

**DOI:** 10.3390/medicina61020245

**Published:** 2025-01-31

**Authors:** Dorde Jevtic, Adela Taylor, Igor Dumic, Erik Sviggum, Charles W. Nordstrom, Marina Antic

**Affiliations:** 1 Icahn School of Medicine at Mount Sinai NYCHHC Elmhurst, Queens, NY 11373, USA; djordje965@gmail.com; 2 Department of Allergy and Immunology, Mayo Clinic Health System, Eau Claire, WI 54703, USA; taylor.adela@mayo.edu; 3 Mayo Clinic College of Medicine and Science, Rochester, MN 55905, USA; dumic.igor@mayo.edu (I.D.); sviggum.erik@mayo.edu (E.S.); nordstrom.cw@mayo.edu (C.W.N.); 4 Department of Hospital Medicine, Mayo Clinic Health System, Eau Claire, WI 54703, USA; 5 Department of Radiology, Mayo Clinic Health System, Eau Claire, WI 54703, USA

**Keywords:** angioedema, C1-inhibitor, gastrointestinal angioedema

## Abstract

*Background and Objectives*: Angioedema is a non-pitting edema of the submucosal layer which can be acquired or inherited and usually presents with hives. Intestinal angioedema is rare and can mimic other acute gastrointestinal disorders. It is typically associated with a lack or dysfunction of C1-inhibitor, with a small number of cases having normal C1-inhibitor function. We present a rare case of chronic recurrent intestinal angioedema in a patient with normal C1-inhibitor function who did not respond to icatibant therapy. *Case presentation*: A 56-year-old woman presented with 3 days of abdominal pain, nausea, vomiting, and diarrhea. She denied a history of allergies and reported a 30-year history of similar episodes requiring hospitalization. Initial evaluation demonstrated normal C4 and C1 esterase inhibitor function with negative gastrointestinal bacterial and viral panel. A CT of the abdomen and pelvis demonstrated acute diffuse bowel thickening and prominent mesenteric lymph nodes. MRI demonstrated inflammation of the small and large bowel. EGD and colonoscopy findings were normal. She was diagnosed with intestinal angioedema and started on icatibant without significant improvement. Her symptoms resolved after 3 days of supportive therapy and resolution of inflammation was noted on imaging. She was discharged home with allergy and immunology follow-up. *Conclusions*: Intestinal angioedema is under-recognized and presentation can overlap with other pathologies of the GI tract. Extensive work up is needed during the first episode of an attack and complement levels should be checked in all patients. Appropriate classification is important as it dictates therapy. However, ambiguous cases like ours sometimes cannot be classified into any specific category.

## 1. Background

Angioedema is a non-pitting edema involving the submucosal layers of the respiratory tract, gastrointestinal tract, and/or skin [1]. It can be classified as either acquired or inherited (HAE), with the estimated prevalence of HAE being 1 in 50,000 [2]. Acquired angioedema usually presents with hives and is associated with type 1 hypersensitivity reactions (HSRs), while HAE involves lack or dysfunction of C1-inhibitor [1,2]. A subset of cases, classified as HAE type 3, exhibit normal C1-inhibitor levels, making it difficult to differentiate them from other forms of idiopathic and acquired angioedema [3,4]. Accurate classification is crucial, as targeted preventative and therapeutic interventions depend on the mediators involved in the specific type of angioedema [4].

Isolated angioedema of the gastrointestinal tract is rare and often under-recognized. It can present with a multitude of symptoms, including abdominal pain, diarrhea, nausea, and vomiting, which may be severe and mimic other gastrointestinal disorders such as peptic ulcer disease, gastroenteritis, inflammatory bowel disease, and acute abdomen. Here, we present a rare case of chronic recurrent intestinal angioedema in a patient with normal complement levels who failed to respond to therapy with icatibant.

## 2. Case Presentation

**Figure 1:**
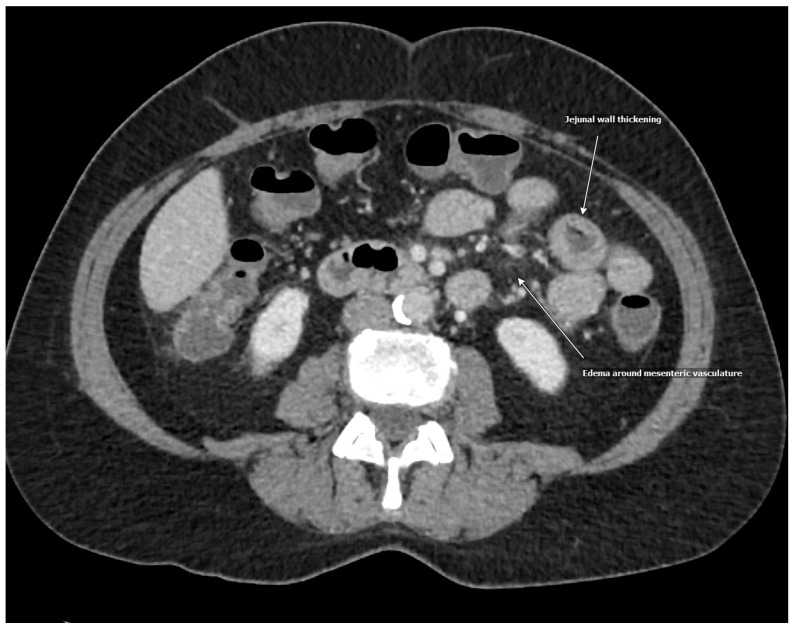
Intestinal angioedema.

**Figure 2:**
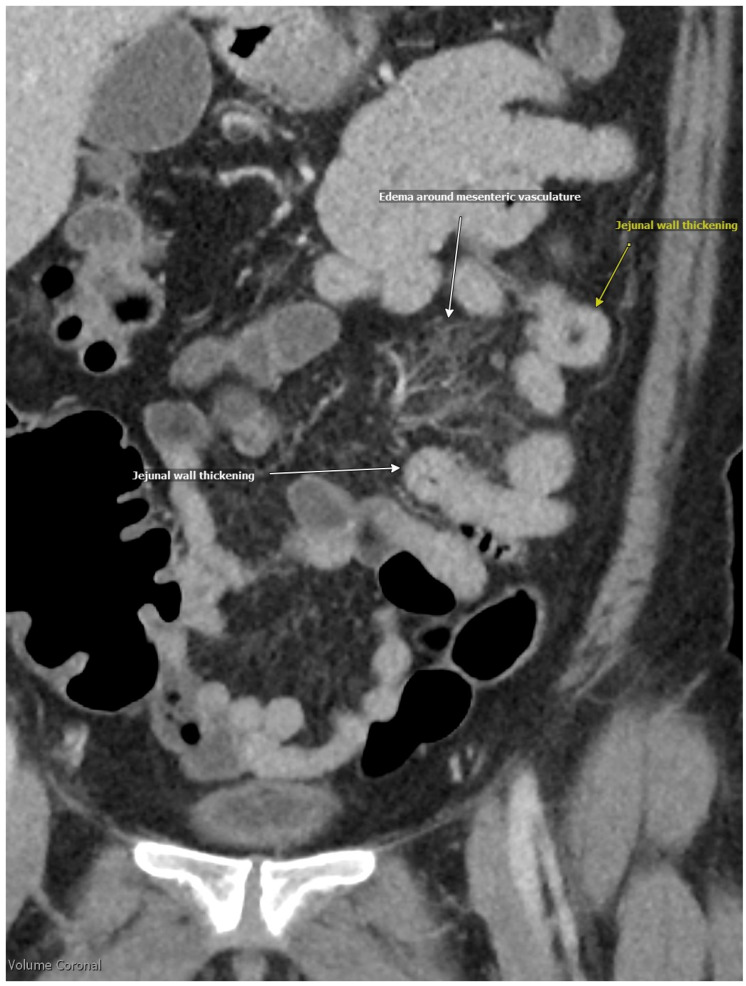
Intestinal angioedema.

**Figure 3:**
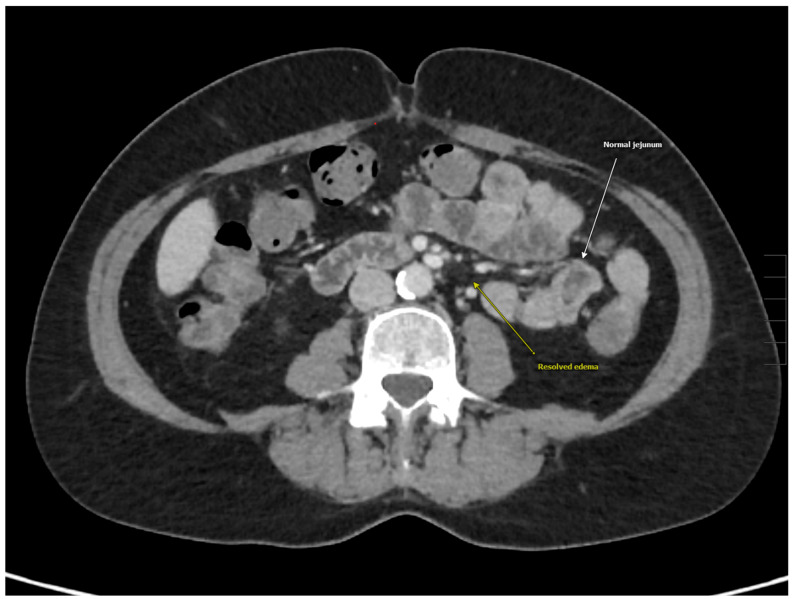
Resolved intestinal angioedema.

A 56-year-old woman with a medical history of dyspepsia, hyperprolactinemia, hypothyroidism, and depression presented to the emergency department with a three-day history of diffuse abdominal pain, non-bloody diarrhea, and one episode of nausea with vomiting. She denied associated symptoms, including fevers, chills, weakness, weight loss, hives, wheezing, congestion, or skin swelling. She also denied a history of allergies, and her family history was negative for any autoimmune, gastrointestinal, or allergic conditions. The patient reported a 30-year history of similar intermittent episodes of abdominal pain, requiring hospitalization every 3–5 years, with the most recent episode being eight years earlier. During that prior hospitalization, she was treated with intravenous fluids and analgesics. On this presentation, her vital signs were stable, and a physical examination revealed generalized abdominal tenderness to palpation. Initial laboratory evaluation demonstrated hemoglobin of 13.3 g/dL (reference range 11.6–15.0 g/dL), leukocytes of 12.4 × 10^9^/L (reference range 3.4–9.6 × 10^9^/L), and chronically elevated platelets at 470 × 10^9^/L (reference range 157–371 × 10^9^ /L). A basic metabolic panel which included electrolytes, blood urea nitrogen, and creatinine, was unremarkable. However, C-reactive protein (CRP) was elevated at 12.5 mL/L (reference range < 7.1 mL/L). Lipase was within normal limits at 21 U/L (reference range 13–60 U/L), and gastrointestinal panel testing was negative for bacterial and viral pathogens. Serologic testing, including rheumatoid factor, anti-neutrophilic cytoplasmic antibody (ANCA), antinuclear antibody (ANA), tissue transglutaminase antibody, and hepatitis serologies, was also negative. A computed tomography (CT) of the abdomen and pelvis with contrast demonstrated acute diffuse bowel wall thickening consistent with pancolitis, ileitis, and peri-appendiceal stranding. Prominent mesenteric lymph nodes were observed in the right lower quadrant, without evidence of intra-abdominal fluid or fatty infiltration along the thickening of the intestinal wall (Figure 1 and Figure 2). The patient received intravenous analgesics and was admitted to inpatient medicine. Magnetic resonance imaging (MRI) of the abdomen demonstrated inflammation of the duodenum, jejunum, and colon, while esophagogastroduodenoscopy (EGD) and colonoscopy were unremarkable. Given her clinical presentation, intestinal angioedema was suspected. Allergy and immunology consultation recommended obtaining complement studies and treatment with icatibant every 8 hours for up to three doses if symptoms persisted after prior doses. Complement studies showed normal levels, including a C4 level of 32 mg/dL (reference range 14–40 mg/dL), C1 esterase inhibitor of 32 mg/dL (reference range 19–37 mg/dL), and C1-esterase inhibitory activity of >90% (reference range > 67%). Due to her long-term history of recurrent attacks of abdominal pain, absence of angiotensin-converting enzyme inhibitor (ACEi), normal complement levels, and negative family history, she was diagnosed with idiopathic angioedema with normal C1-inhibitor function. Her symptoms did not improve significantly after icatibant administration; however, they eventually resolved over the course of 3 days with pain medication and fluids. Repeat CT scan demonstrated complete resolution of bowel wall thickening and lymphadenopathy (Figure 3). The patient’s hospital course was unremarkable, and she was discharged home with an outpatient allergy and immunology follow-up.

## 3. Discussion and Conclusions

### 3.1. Genetics, Pathophysiology, and Classification of Angioedema

Angioedema is usually driven by histamine or bradykinin mediators and can be hereditary or nonhereditary/acquired. Histamine-mediated angioedema is caused by IgE-mediated degranulation of mast cells (type I HSRs) or is a consequence of direct mastocyte degranulation (e.g., iodine/gadolinium contrast) [5]. HAE is an autosomal dominant genetic disorder that usually manifests in childhood [4]. The mutations involve the *SERPING1* gene resulting in deficient (type 1) or non-functional (type 2) C1-inhibitor [4]. C1-inhibitor is the main inhibitor of the kallikrein-kinin system. Due to a lack of inhibition, bradykinin production is increased, which mediates vasodilatation, angiogenesis, and edema. A small number of patients have HAE type 3, which is likely under-represented in the literature and was first described in the 2000s in a set of 10 families and 36 female patients with normal levels and activity of C1-inhibitor [3]. To date, six mutations have been identified as underlying causes of hereditary angioedema in patients with normal C1-inhibitor levels [6,7]. Understanding these genetic factors is crucial for accurately diagnosing the condition using next-generation sequencing technologies [8]. These technologies have facilitated the identification of mutations in five genes: ANGPT1, PLG, KNG1, MYOG, and HS3ST6 [8]. In 2006, Dewald and Bork identified one of the first distinct missense mutations resulting in the substitution of threonine with lysine or arginine. This mutation produces a truncated factor XII, leading to increased bradykinin production [9]. Notably, these mutations were more prevalent in female patients, particularly during periods of heightened estrogen levels, such as pregnancy or contraceptive use [10,11]. In 2018, Baffuno et al. described a mutation in the angiopoietin-1 gene (ANGPT1) which contributes to increased vascular permeability and angioedema [12]. Similarly, Ariano et al. identified a gain-of-function mutation in the MYOF gene, also linked to enhanced vascular permeability [13]. Another important discovery was a missense mutation in the plasminogen gene (PLG), identified in 18 patients, which results in a lysine-to-glutamic acid substitution predisposing individuals to angioedema [14]. Most recently, in 2021, Brok et al. described a novel mutation in the HS3ST6 gene, which encodes heparan sulfate 3-O-sulfotransferase 6 contributing to recurrent angioedema [15]. Advances in genetic technologies are expected to uncover additional genes and mutations associated with hereditary angioedema in patients with normal C1-inhibitor levels, offering new opportunities for personalized and targeted therapies. Another form of acquired bradykinin-mediated angioedema is associated with ACEi use, typically diagnosed based on clinical history [16]. Other less common forms include drug-induced angioedema (e.g., nonsteroid anti-inflammatory drugs (NSAIDs), statins) and idiopathic angioedema, for which the mediators remain unknown [17,18]. A limitation of this report is the absence of genetic studies, which may have provided valuable insights into the underlying pathophysiology and facilitated a more precise diagnosis.

While establishing diagnosis in our patient was difficult, we reached this by eliminating other potential causes and obtaining results of extensive work-up including CTAP, abdominal MRI, EGD, colonoscopy, infectious, and autoimmune studies (Table 1). Our patient presented with decades-long recurrent angioedema, which is typically seen in hereditary forms. However, her family history was negative. She had no extra intestinal symptoms, hives, or pruritus, which would be present in a histamine-mediated process. There were no underlying causes or triggers for her intestinal edema. Finally, she failed to respond appropriately to a bradykinin antagonist, which could rule out the bradykinin-mediated process [4]. However, the timing of medication initiation three days after the onset of her symptoms likely precluded an appropriate response [17]. Genetic tests were not carried out, and it could be postulated that she experienced a de novo mutation resulting in a presentation similar to HAE type 3. Prior studies demonstrated an equivocal utility of genetic testing in HAE, especially type 3 [19]. She would not meet the criteria for idiopathic angioedema as her attacks occurred every few years compared to the idiopathic angioedema which requires ≥3 attacks in a 6–12 month period [19]. In cases of acquired angioedema which occurs later in life, the complement studies are typically decreased, unlike our patient who had normal complement levels. In summary, our patient does not meet criteria for any of the angioedema groups, based on the current classification system.

### 3.2. Clinical Presentation, Symptoms, and Laboratory

Angioedema often presents dramatically with sudden onset of localized and often asymmetric swelling of the subcutaneous and any submucosal tissue, including skin, upper airway, and gastrointestinal tract. While typically self-limiting, resolving within a few hours to days, prompt recognition and treatment are crucial due its potential to become life-threatening. It frequently causes asymmetric skin and mucous membrane swelling and may be accompanied by urticaria [20]. Histaminergic-mediated angioedema is associated with urticaria in approximately 40% of cases [21]. Conversely, angioedema without urticaria is less common, occurring in about 10% of cases with idiopathic acquired angioedema [22]. The unpredictable nature of angioedema significantly impacts the patients’ quality of life, often causing anxiety, fear, and frustration, particularly when the triggers for attacks remain unidentified [20]. Previous reports have demonstrated that some patients present with atypical symptoms, such as episodic severe abdominal pain without mucocutaneous swelling in other body regions [23]. This can complicate the diagnosis and delay appropriate treatment [23]. Additional symptoms may include nausea, vomiting, diarrhea, abdominal distension, and ascites, which result from intestinal swelling and fluid shifts between the intestinal lumen and peritoneal cavity [24]. In severe cases, the symptoms can mimic an acute abdomen with rigidity and abdominal guarding, occasionally leading to unnecessary invasive surgical procedures [22]. Initial evaluation should focus on determining the location, timing, duration, and circumstances when the swelling occurs to identify a potential trigger.

Laboratory workup should be individualized, but often includes a complete blood count with differential, basic metabolic panel, liver function tests, CRP, thyroid stimulation hormone (TSH), tryptase, and IgE [20]. When hereditary angioedema or acquired C1-inhibitor deficiency is suspected, testing should include C1-inhibitor concentration and function, as well as C4 and C1q levels. C4 is commonly used as a screening test, as it is decreased during attacks of HAE types 1 and 2 but can normalize between episodes. In contrast, C4 levels are typically normal in HAE type 3 [2]. C1q is particularly useful in cases of acquired angioedema, as it is decreased in these clinical situations, but remains normal in HAE [2]. Our patient’s normal C4 and C1-inhibitor levels ruled out HAE types 1 and 2 and acquired angioedema. Imaging studies are helpful in diagnosing intestinal angioedema and can reveal characteristic findings such as thickened small bowel walls, mucosal edema with prominent vessels, ascites, and fluid accumulation [25]. Prior studies have demonstrated that 90% of the patients will present with free fluid in the abdominal cavity, a finding which was not observed in our patient [26]. Repeat imaging after the resolution of symptoms shows normalization of bowel structure [25,26], a pattern of dynamic change consistent with our patient’s clinical course. Endoscopy is not recommended in the acute setting due to the risk of inducing life-threatening laryngeal edema which may necessitate emergent intubation [17].

## 4. Treatment and Outcome

Treatment of angioedema focuses on acute management and prophylaxis, tailored to the underlying mechanism. In histamine-driven angioedema, anaphylaxis protocols should be implemented, as these are commonly life-threatening [19]. Epinephrine is the first-line treatment and should be administered without delay, along with adjunctive therapies such as antihistamines and steroids [19]. Patients should be provided with epinephrine auto-injectors for emergency use at home in case of future exposure to known triggers. Emerging evidence suggests that Omalizumab, which binds free IgE and decreases the expression of high-affinity IgE receptors, may serve as an effective adjunctive therapy in histaminergic angioedema [27]. For bradykinin-mediated angioedema, first-line acute therapies include ecallantide, icatibant, human plasma, and recombinant C1-inhibitor [2]. Ecallantide, a plasma kallikrein inhibitor, reduces bradykinin production, while icatibant acts as a bradykinin receptor antagonist. Both therapies have a short half-life and are effective in managing acute attacks [2]. C1-inhibitors, which replace dysfunctional or deficient C1-inhibitor proteins, have longer half-lives and can be used both acutely and as prophylaxis [2]. Our patient was treated with icatibant but did not demonstrate a clinically significant response with symptoms persisting for several days after administration. This diminished efficacy could be attributed to a delay in administration, as icatibant is most effective when given within hours of symptom onset [28]. Additionally, icatibant can be self-administered at home and has been proven to shorten the duration of attacks and expedite treatment initiation [29].

Intestinal angioedema should be suspected in all patients who present with gastrointestinal symptoms, as this condition is commonly under-recognized. Due to significant overlap with other gastrointestinal pathologies, an extensive work-up is warranted during the first episode of symptoms. For subsequent episodes, early treatment is pivotal in order to mitigate symptom progression. Complement levels should be checked in all patients at baseline and during the attacks to aid diagnosis, and in some cases, genetic testing is necessary to elucidate the diagnosis. Classification of angioedema is essential for guiding treatment; however, cases like that of our patient highlight the complexities of diagnosis and management. Such ambiguity poses challenges for clinicians both in the acute and the outpatient settings. Further research is needed to refine therapeutic algorithms, particularly for patients with isolated gastrointestinal angioedema that is not mediated by type 1 HSRs and does not respond to bradykinin antagonists. A revision of the classification system might be necessary in order to include ambiguous cases, such as the one described in this case report.

## Figures and Tables

**Table 1:** Diagnostic work-up.

Studies	Results
WBC	12.4 × 10^9^/L (reference range 3.4–9.6 × 10^9^/L)
Infectious work up	GI and respiratory panel negative, UA unremarkable
CRP	12.5 mL/L (reference range < 7.1 mL/L).
CTAP	Pancolitis, ileitis, and peri-appendiceal stranding with prominent right lower quadrant mesenteric lymph nodes
MRI	Inflammation of the duodenum, jejunum, and colon
EGD/Colonoscopy	Unremarkable
Complement studies	C4: 32 mg/dL (reference range 14–40 mg/dL) C1 esterase inhibitor: 32 mg/dL (reference range 19–37 mg/dL).

WBC-White blood cells, GI-Gastrointestinal, UA-Urinalysis, CRP-C-reactive protein, CTAP-Computed tomography abdomen and pelvis, MRI-Magnetic resonance imaging, EGD-Esophagogastroduodenoscopy.

## Data Availability

Data are contained within the article.
